# The β-modification of trizinc borate phosphate, Zn_3_(BO_3_)(PO_4_)

**DOI:** 10.1107/S1600536810051871

**Published:** 2010-12-24

**Authors:** Erpan Zhang, Sangen Zhao, Jianxiu Zhang, Peizhen Fu, Jiyong Yao

**Affiliations:** a Key Laboratory of Functional Crystal and Laser Technology, Technical Institute of Physics and Chemistry, Chinese Academy of Sciences, Beijing 100190, People’s Republic of China; b Graduate University of Chinese Academy of Sciences, Beijing 100049, People’s Republic of China

## Abstract

Crystals of β-Zn_3_(BO_3_)(PO_4_) have been grown by the Kyropoulos method. The asymmetric unit contains three Zn sites, three B-atom sites (all with symmetry 3), two P sites (both with *m* symmetry) and nine O-atom sites (four with *m* symmetry). The fundamental building units of the title structure are isolated BO_3_ triangles and PO_4_ tetra­hedra, which are bridged by ZnO_4_ tetra­hedra or ZnO_5_ trigonal bipyramids through common O atoms, leading to a three-dimensional framework structure. Some significant structural differences between the β-polymorph and the α-polymorph are discussed.

## Related literature

For general backround to Zn_3_(BO_3_)(PO_4_), see: Liebertz & Stahr (1982 ▶). For crystal growth of β-Zn_3_(BO_3_)(PO_4_), see: Wang *et al.* (2000 ▶); Wu & Wang (2001 ▶); Liu *et al.* (2002 ▶). For structure refinement of α-Zn_3_(BO_3_)(PO_4_), see: Bluhm & Park (1997 ▶). For structurally related compounds, see: Ma *et al.* (2004 ▶); Yilmaz *et al.* (2001 ▶). Reviews on borophosphates were given by Kniep *et al.* (1998 ▶) and Ewald *et al.* (2007 ▶).

## Experimental

### 

#### Crystal data

Zn_3_(BO_3_)(PO_4_)
*M* *_r_* = 349.89
Hexagonal, 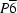

*a* = 8.4624 (3) Å
*c* = 13.0690 (7) Å
*V* = 810.51 (4) Å^3^
*Z* = 6
Mo *K*α radiation
μ = 13.49 mm^−1^
*T* = 294 K
0.25 × 0.22 × 0.16 mm

#### Data collection

Rigaku Saturn CCD diffractometer
Absorption correction: numerical (*NUMABS*; Rigaku, 2005 ▶) *T*_min_ = 0.133, *T*_max_ = 0.221
10584 measured reflections
1453 independent reflections
1226 reflections with *I* > 2σ(*I*)
*R*_int_ = 0.062

#### Refinement

*R*[*F* ^2^ > 2σ(*F* ^2^)] = 0.019
*wR*(*F* ^2^) = 0.039
*S* = 0.96
1453 reflections
118 parameters
Δρ_max_ = 0.61 e Å^−3^
Δρ_min_ = −0.52 e Å^−3^
Absolute structure: Flack (1983 ▶), 718 Friedel pairs
Flack parameter: 0.011 (10)

### 

Data collection: *CrystalClear* (Rigaku, 2005 ▶); cell refinement: *CrystalClear*; data reduction: *CrystalClear*; program(s) used to solve structure: *SHELXS97* (Sheldrick, 2008 ▶); program(s) used to refine structure: *SHELXL97* (Sheldrick, 2008 ▶); molecular graphics: *DIAMOND* (Brandenburg, 2006 ▶); software used to prepare material for publication: *publCIF* (Westrip, 2010 ▶).

## Supplementary Material

Crystal structure: contains datablocks I, global. DOI: 10.1107/S1600536810051871/wm2419sup1.cif

Structure factors: contains datablocks I. DOI: 10.1107/S1600536810051871/wm2419Isup2.hkl

Additional supplementary materials:  crystallographic information; 3D view; checkCIF report

## Figures and Tables

**Table 1:** Selected bond lengths (Å)

Zn1—O7	1.9248 (17)
Zn1—O2^i^	1.9293 (19)
Zn1—O3^ii^	2.084 (2)
Zn1—O1	2.100 (2)
Zn2—O7	1.9529 (17)
Zn2—O9^iii^	1.9604 (18)
Zn2—O8^iv^	1.9656 (17)
Zn2—O4	2.2019 (18)
Zn2—O2	2.3238 (18)
Zn3—O4^v^	1.9958 (18)
Zn3—O8	2.035 (2)
Zn3—O5	2.0704 (18)
Zn3—O6^vi^	2.0748 (18)
Zn3—O9	2.079 (2)
B1—O7	1.3792 (18)
B2—O8	1.3880 (18)
B3—O9	1.3775 (17)
P1—O3	1.541 (3)
P1—O1	1.542 (3)
P1—O2	1.5484 (18)
P2—O5	1.529 (3)
P2—O6	1.540 (3)
P2—O4	1.5412 (18)

Symmetry codes: (i) 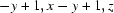; (ii) 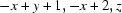; (iii) 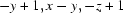; (iv) 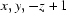; (v) 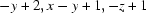; (vi) 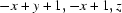.

**Fig. 1.**
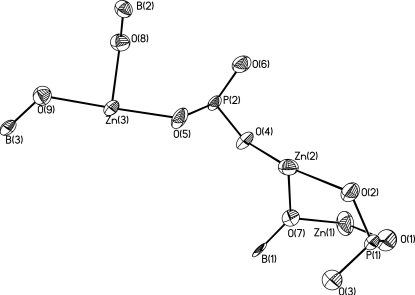
The asymmetric unit of β-Zn3(BO3)(PO4) with atom labelling and ellipsoids drawn at the 90% probability level.

**Fig. 2.**
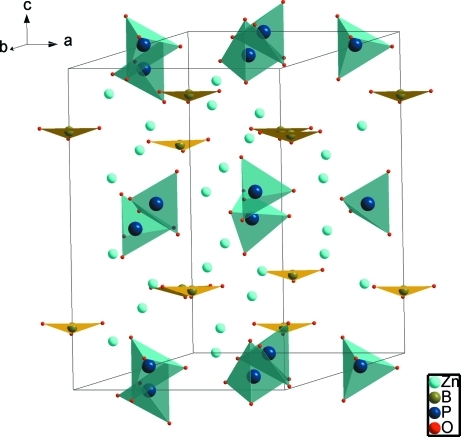
Crystal structure of β-Zn3(BO3)(PO4) illustrated with isolated BO3 and PO4 groups.

**Fig. 3.**
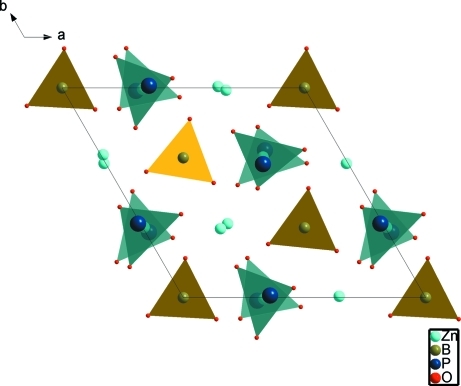
The structure of β-Zn3(BO3)(PO4) viewed along [001].

## supplementary crystallographic information

### Comment

Liebertz & Stahr reported the existence of Zn_3_(BO_3_)(PO_4_) in 1982 (Liebertz & Stahr, 1982). Zn_3_(BO_3_)(PO_4_) (ZBP) can exist in two modifications, one low-temperature phase denoted α and one high-temperature phase denoted β. The phase transition point is 875 K. Some significant features of β-Zn_3_(BO_3_)(PO_4_) make it attractive as a promising NLO material. However, β-Zn_3_(BO_3_)(PO_4_) crystals grown from the melt frequently have a poor quality when cooling to room temperature. Hence considerable effort has been made to obtain high-quality β-Zn_3_(BO_3_)(PO_4_) crystals (Wang *et al.*, 2000; Wu & Wang, 2001; Liu *et al.*, 2002). In this paper, β-Zn_3_(BO_3_)(PO_4_) crystals were obtained through a rapid cooling method. The structural differences between α-Zn_3_(BO_3_)(PO_4_) and β-Zn_3_(BO_3_)(PO_4_) are described, and we also briefly discuss the structural differences between β-Zn_3_(BO_3_)(PO_4_) and the structures of other borate-phosphates and borophosphates.

The asymmetric unit of the title structure contains three Zn sites, three B sites (all with site symmetry 3), two P sites (both with *m* symmetry) and nine O sites (four of which with *m* symmetry) (Fig. 1). The fundamental structural building units are isolated BO_3_ triangles and PO_4_ tetrahedra. These units are alternately oriented parallel to (001) and stacked layer upon layer along [001] (Figs. 2 and 3). The isolated character of the anionic units classifies this compound as a borate-phosphate in contrast to borophosphates, where at least one BO_3_ (or BO_4_) group and one PO_4_ tetrahedron share a common O atom. Reviews on the crystal chemistry of the latter class of compounds were given by Kniep *et al.* (1998) and Ewald *et al.* (2007).

The BO_3_ triangles show an equilateral trigonal-planar configuration. The P—O distances range between 1.529 (3) Å and 1.5484 (18) Å, indicating a slight distortion of the two PO_4_ tetrahedra in this structure. The Zn atoms selectively occupy the space between the anionic layers and are bonded to the terminal O atoms of the anions. The coordination environments of the three independent Zn are different from another. Zn1 is tetrahedrally surrounded by atoms O7, O2, O3 and O1, with Zn—O distances in the range 1.9248 (17) Å – 2.100 (2) Å. Zn2 and Zn3 are five-coordinate by oxygen within a trigonal bipyramid and Zn—O distances range from 1.9529 (17) Å to 2.3238 (18) Å for Zn2 and from 1.9958 (17) Å to 2.079 (2) Å for Zn3. The isolated BO_3_ and PO_4_ groups are linked to ZnO_5_ or ZnO_4_ polyhedra by sharing one corner O atom. In addition, ZnO_5_ and ZnO_4_ polyhedra are linked together by sharing one corner O atom. Individual ZnO_4_ tetrahedra and ZnO_5_ polyhedra, respectively, also share a common edge.

To the best of our knowledge, besides β-Zn_3_(BO_3_)(PO_4_), α-Zn_3_(BO_3_)(PO_4_) (Bluhm & Park, 1997), Co_3_(BO_3_)(PO_4_) (Yilmaz *et al.*, 2001) and Ba_3_(BO_3_)(PO_4_) (Ma *et al.*., 2004) are the only three other borate-phosphates. In comparison with β-Zn_3_(BO_3_)(PO_4_), in the structure of α-Zn_3_(BO_3_)(PO_4_) the BO_3_ and PO_4_ groups are also linked to ZnO_5_ trigonal bipyramids and ZnO_4_ tetrahedra by sharing O atoms. However, the symmetry of the two structures is different. During the α→β phase transformation, the positions of the isolated BO_3_ and PO_4_ groups are rearranged, accompanied with a change from space group *Cm* (α-phase) to *P*6 (β-phase). The density of low-temperature α-Zn_3_(BO_3_)(PO_4_) is 4.44 g/cm^3^ (Bluhm & Park, 1997), while the density of high-temperature β-Zn_3_(BO_3_)(PO_4_) is 4.30 g/cm^3^, pointing to α-Zn_3_(BO_3_)(PO_4_) as the thermodynamically stable phase. It should be noted that the β-Zn_3_(BO_3_)(PO_4_) structure type is different from Ba_3_(BO_3_)(PO_4_) (space group *P*6_3_*mc*) whereas Co_3_(BO_3_)(PO_4_) is isotypic with α-Zn_3_(BO_3_)(PO_4_). The differences between the Ba-containing structure and the structures containing the first row-transition metals is caused by the different ionic radii of the metal cations and consequently by a different coordination environment.

### Experimental

Zn_3_(BO_3_)(PO_4_) was synthesized by a standard solid-state reaction of the starting components, using chemically pure ZnO, H_3_BO_3_ and NH_4_H_2_PO_4_ in the molar ratio of 3:1:1. A platinum crucible filled with Zn_3_(BO_3_)(PO_4_) was heated to 1273 K, kept at that temperature for 12 h, and then was cooled to the saturation temperature. A seed crystal of β-Zn_3_(BO_3_)(PO_4_) attached to a platinum rod was inserted into the solution, and then the temperature was cooled at a rate of 0.3 K^.^d^-1^ until the end of the growth. The obtained crystal was pulled out of the surface of the solution, cooled to 700 K at a rate of 20 K/h, and then cooled rapidly to 620 K in 1.5 h. Finally, the crystal was removed out of the furnace and cooled to room temperature.

### Refinement

One B atom (B1) has been refined with an isotropic displacement parameter. Refinement with anisotropic displacement parameters for this atom resulted in physically meaningless values.

### Crystal data

**Table:**

Zn_3_(BO_3_)(PO_4_)	*D*_x_ = 4.301 Mg m^−^^3^
*M**_r_* = 349.89	Melting point: 1200 K
Hexagonal, *P*6	Mo *K*α radiation, λ = 0.71073 Å
Hall symbol: -P 6	Cell parameters from 2470 reflections
*a* = 8.4624 (3) Å	θ = 1.6–28.7°
*c* = 13.0690 (7) Å	µ = 13.49 mm^−^^1^
*V* = 810.51 (4) Å^3^	*T* = 294 K
*Z* = 6	Prism, colorless
*F*(000) = 996	0.25 × 0.22 × 0.16 mm

### Data collection

**Table:**

Rigaku Saturn CCD diffractometer	1453 independent reflections
Radiation source: fine-focus sealed tube	1226 reflections with *I* > 2σ(*I*)
confocal	*R*_int_ = 0.062
Detector resolution: 7.31 pixels mm^-1^	θ_max_ = 28.6°, θ_min_ = 1.6°
ω scans	*h* = −11→10
Absorption correction: numerical (*NUMABS*; Rigaku, 2005)	*k* = −11→11
*T*_min_ = 0.133, *T*_max_ = 0.221	*l* = −17→17
10584 measured reflections	

### Refinement

**Table:**

Refinement on *F*^2^	Secondary atom site location: difference Fourier map
Least-squares matrix: full	*w* = 1/[σ^2^(*F*_o_^2^) + (0.0099*P*)^2^] where *P* = (*F*_o_^2^ + 2*F*_c_^2^)/3
*R*[*F*^2^ > 2σ(*F*^2^)] = 0.019	(Δ/σ)_max_ = 0.004
*wR*(*F*^2^) = 0.039	Δρ_max_ = 0.61 e Å^−^^3^
*S* = 0.96	Δρ_min_ = −0.52 e Å^−^^3^
1453 reflections	Extinction correction: *SHELXL97* (Sheldrick, 2008), Fc^*^=kFc[1+0.001xFc^2^λ^3^/sin(2θ)]^-1/4^
118 parameters	Extinction coefficient: 0.0328 (7)
0 restraints	Absolute structure: Flack (1983), 718 Friedel pairs
0 constraints	Flack parameter: 0.011 (10)
Primary atom site location: structure-invariant direct methods	

### Special details

**Table:**

Geometry. All e.s.d.'s (except the e.s.d. in the dihedral angle between two l.s. planes) are estimated using the full covariance matrix. The cell e.s.d.'s are taken into account individually in the estimation of e.s.d.'s in distances, angles and torsion angles; correlations between e.s.d.'s in cell parameters are only used when they are defined by crystal symmetry. An approximate (isotropic) treatment of cell e.s.d.'s is used for estimating e.s.d.'s involving l.s. planes.
Refinement. Refinement of *F*^2^ against ALL reflections. The weighted *R*-factor *wR* and goodness of fit *S* are based on *F*^2^, conventional *R*-factors *R* are based on *F*, with *F* set to zero for negative *F*^2^. The threshold expression of *F*^2^ > σ(*F*^2^) is used only for calculating *R*-factors(gt) *etc*. and is not relevant to the choice of reflections for refinement. *R*-factors based on *F*^2^ are statistically about twice as large as those based on *F*, and *R*- factors based on ALL data will be even larger.

### Fractional atomic coordinates and isotropic or equivalent isotropic displacement parameters (Å^2^)

**Table:**

	*x*	*y*	*z*	*U*_iso_*/*U*_eq_	
Zn1	0.67111 (7)	0.98916 (9)	0.88495 (3)	0.01824 (11)	
Zn2	0.66452 (5)	0.67089 (5)	0.74557 (2)	0.01218 (11)	
Zn3	0.99324 (6)	0.64008 (5)	0.38229 (3)	0.00982 (9)	
B1	1.0000	1.0000	0.8052 (5)	0.0097 (11)*	
B2	0.6667	0.3333	0.2820 (5)	0.0110 (12)	
B3	1.3333	0.6667	0.2751 (4)	0.0088 (11)	
P1	0.64705 (15)	0.63672 (14)	1.0000	0.0085 (2)	
P2	0.68800 (15)	0.70242 (14)	0.5000	0.0083 (2)	
O1	0.6018 (3)	0.7921 (4)	1.0000	0.0140 (6)	
O2	0.5676 (3)	0.5214 (2)	0.90172 (13)	0.0137 (4)	
O3	0.8535 (3)	0.7058 (4)	1.0000	0.0146 (7)	
O4	0.7144 (2)	0.8140 (2)	0.59811 (13)	0.0116 (4)	
O5	0.8298 (4)	0.6407 (4)	0.5000	0.0141 (6)	
O6	0.4931 (4)	0.5371 (4)	0.5000	0.0135 (6)	
O7	0.8121 (2)	0.9147 (2)	0.80352 (13)	0.0116 (4)	
O8	0.7773 (3)	0.5217 (2)	0.28461 (17)	0.0123 (5)	
O9	1.1480 (2)	0.6008 (2)	0.27238 (16)	0.0124 (4)	

### Atomic displacement parameters (Å^2^)

**Table:**

	*U*^11^	*U*^22^	*U*^33^	*U*^12^	*U*^13^	*U*^23^
Zn1	0.0165 (2)	0.0204 (2)	0.0210 (2)	0.01163 (17)	0.00515 (18)	−0.00218 (19)
Zn2	0.0110 (2)	0.01191 (19)	0.0137 (2)	0.00580 (16)	−0.00283 (15)	−0.00386 (13)
Zn3	0.0110 (2)	0.01235 (19)	0.00559 (17)	0.00543 (19)	−0.00002 (14)	−0.00152 (13)
B2	0.0095 (18)	0.0095 (18)	0.014 (3)	0.0048 (9)	0.000	0.000
B3	0.0120 (17)	0.0120 (17)	0.002 (3)	0.0060 (9)	0.000	0.000
P1	0.0091 (5)	0.0101 (5)	0.0051 (5)	0.0039 (5)	0.000	0.000
P2	0.0109 (6)	0.0114 (5)	0.0035 (5)	0.0064 (5)	0.000	0.000
O1	0.0167 (15)	0.0155 (15)	0.0142 (17)	0.0112 (13)	0.000	0.000
O2	0.0136 (10)	0.0141 (10)	0.0083 (10)	0.0029 (9)	0.0011 (8)	−0.0003 (8)
O3	0.0118 (14)	0.0173 (15)	0.0125 (17)	0.0058 (12)	0.000	0.000
O4	0.0175 (11)	0.0137 (10)	0.0060 (10)	0.0096 (9)	−0.0005 (8)	−0.0016 (8)
O5	0.0191 (15)	0.0243 (16)	0.0047 (16)	0.0153 (13)	0.000	0.000
O6	0.0146 (15)	0.0137 (15)	0.0075 (16)	0.0036 (12)	0.000	0.000
O7	0.0119 (9)	0.0123 (10)	0.0100 (11)	0.0056 (8)	−0.0005 (8)	−0.0012 (8)
O8	0.0122 (10)	0.0120 (10)	0.0141 (13)	0.0072 (8)	−0.0026 (9)	−0.0014 (9)
O9	0.0128 (10)	0.0109 (10)	0.0137 (11)	0.0060 (9)	0.0004 (9)	−0.0010 (9)

### Geometric parameters (Å, °)

**Table:**

Zn1—O7	1.9248 (17)	B1—O7^ii^	1.3792 (18)
Zn1—O2^i^	1.9293 (19)	B1—O7	1.3792 (18)
Zn1—O3^ii^	2.084 (2)	B1—O7^ix^	1.3792 (18)
Zn1—O1	2.100 (2)	B2—O8^vii^	1.3880 (18)
Zn1—Zn1^iii^	3.0071 (9)	B2—O8	1.3880 (18)
Zn2—O7	1.9529 (17)	B2—O8^x^	1.3880 (18)
Zn2—O9^iv^	1.9604 (18)	B3—O9	1.3775 (17)
Zn2—O8^v^	1.9656 (17)	B3—O9^xi^	1.3775 (17)
Zn2—O4	2.2019 (18)	B3—O9^xii^	1.3775 (17)
Zn2—O2	2.3238 (18)	P1—O3	1.541 (3)
Zn2—Zn3^iv^	3.1202 (5)	P1—O1	1.542 (3)
Zn3—O4^vi^	1.9958 (18)	P1—O2	1.5484 (18)
Zn3—O8	2.035 (2)	P1—O2^iii^	1.5484 (18)
Zn3—O5	2.0704 (18)	P2—O5	1.529 (3)
Zn3—O6^vii^	2.0748 (18)	P2—O6	1.540 (3)
Zn3—O9	2.079 (2)	P2—O4	1.5412 (18)
Zn3—Zn3^v^	3.0766 (7)	P2—O4^v^	1.5412 (18)
Zn3—Zn2^viii^	3.1202 (5)		
			
O7—Zn1—O2^i^	152.13 (8)	O1—P1—O2	108.83 (10)
O7—Zn1—O3^ii^	103.27 (9)	O3—P1—O2^iii^	106.96 (10)
O2^i^—Zn1—O3^ii^	101.65 (9)	O1—P1—O2^iii^	108.83 (10)
O7—Zn1—O1	96.22 (8)	O2—P1—O2^iii^	112.09 (15)
O2^i^—Zn1—O1	100.50 (9)	O5—P2—O6	110.90 (15)
O3^ii^—Zn1—O1	79.32 (8)	O5—P2—O4	108.15 (9)
O7—Zn2—O9^iv^	124.56 (7)	O6—P2—O4	108.54 (9)
O7—Zn2—O8^v^	119.80 (8)	O5—P2—O4^v^	108.15 (9)
O9^iv^—Zn2—O8^v^	115.29 (7)	O6—P2—O4^v^	108.54 (9)
O7—Zn2—O4	85.00 (7)	O4—P2—O4^v^	112.59 (15)
O9^iv^—Zn2—O4	92.42 (7)	P1—O1—Zn1^iii^	125.99 (8)
O8^v^—Zn2—O4	99.02 (8)	P1—O1—Zn1	125.99 (8)
O7—Zn2—O2	95.72 (7)	Zn1^iii^—O1—Zn1	91.46 (11)
O9^iv^—Zn2—O2	79.41 (8)	P1—O2—Zn1^xiii^	124.98 (11)
O8^v^—Zn2—O2	88.80 (8)	P1—O2—Zn2	117.49 (10)
O4—Zn2—O2	170.57 (7)	Zn1^xiii^—O2—Zn2	107.41 (8)
O4^vi^—Zn3—O8	131.36 (8)	P1—O3—Zn1^xiv^	128.42 (8)
O4^vi^—Zn3—O5	94.60 (9)	P1—O3—Zn1^ix^	128.42 (8)
O8—Zn3—O5	91.76 (8)	Zn1^xiv^—O3—Zn1^ix^	92.36 (11)
O4^vi^—Zn3—O6^vii^	103.14 (9)	P2—O4—Zn3^xv^	126.50 (11)
O8—Zn3—O6^vii^	125.21 (9)	P2—O4—Zn2	117.50 (10)
O5—Zn3—O6^vii^	76.73 (8)	Zn3^xv^—O4—Zn2	106.43 (8)
O4^vi^—Zn3—O9	91.86 (7)	P2—O5—Zn3^v^	129.80 (7)
O8—Zn3—O9	88.31 (8)	P2—O5—Zn3	129.80 (7)
O5—Zn3—O9	171.33 (10)	Zn3^v^—O5—Zn3	95.98 (11)
O6^vii^—Zn3—O9	96.17 (8)	P2—O6—Zn3^x^	127.95 (8)
O7^ii^—B1—O7	119.976 (16)	P2—O6—Zn3^iv^	127.95 (8)
O7^ii^—B1—O7^ix^	119.976 (16)	Zn3^x^—O6—Zn3^iv^	95.71 (11)
O7—B1—O7^ix^	119.976 (16)	B1—O7—Zn1	124.1 (2)
O8^vii^—B2—O8	119.94 (3)	B1—O7—Zn2	121.32 (16)
O8^vii^—B2—O8^x^	119.94 (3)	Zn1—O7—Zn2	112.75 (9)
O8—B2—O8^x^	119.94 (3)	B2—O8—Zn2^v^	117.88 (13)
O9—B3—O9^xi^	119.93 (2)	B2—O8—Zn3	120.3 (2)
O9—B3—O9^xii^	119.93 (2)	Zn2^v^—O8—Zn3	114.50 (9)
O9^xi^—B3—O9^xii^	119.93 (2)	B3—O9—Zn2^viii^	112.75 (11)
O3—P1—O1	113.22 (15)	B3—O9—Zn3	126.7 (2)
O3—P1—O2	106.96 (10)	Zn2^viii^—O9—Zn3	101.09 (8)

Symmetry codes: (i) −*y*+1, *x*−*y*+1, *z*; (ii) −*x*+*y*+1, −*x*+2, *z*; (iii) *x*, *y*, −*z*+2; (iv) −*y*+1, *x*−*y*, −*z*+1; (v) *x*, *y*, −*z*+1; (vi) −*y*+2, *x*−*y*+1, −*z*+1; (vii) −*x*+*y*+1, −*x*+1, *z*; (viii) −*x*+*y*+1, −*x*+1, −*z*+1; (ix) −*y*+2, *x*−*y*+1, *z*; (x) −*y*+1, *x*−*y*, *z*; (xi) −*x*+*y*+2, −*x*+2, *z*; (xii) −*y*+2, *x*−*y*, *z*; (xiii) −*x*+*y*, −*x*+1, *z*; (xiv) −*y*+2, *x*−*y*+1, −*z*+2; (xv) −*x*+*y*+1, −*x*+2, −*z*+1.

## References

[bb1] Bluhm, K. & Park, C. H. (1997). *Z. Naturforsch. Teil B*, **52**, 102-106.

[bb2] Brandenburg, K. (2006). *DIAMOND* Crystal Impact GbR, Bonn, Germany.

[bb3] Ewald, B., Huang, Y. X. & Kniep, R. (2007). *Z. Anorg. Allg. Chem.* **633**, 1517–1540.

[bb4] Flack, H. D. (1983). *Acta Cryst.* A**39**, 876–881.

[bb5] Kniep, R., Engelhardt, H. & Hauf, C. (1998). *Chem. Mater.* **10**, 2930–2934.

[bb6] Liebertz, J. & Stahr, S. (1982). *Z. Kristallogr.* **160**, 135–137.

[bb7] Liu, H. J., Wu, Y. C. & Wang, G. F. (2002). *J. Synth. Cryst* **31**, 81-84.

[bb8] Ma, H. W., Liang, J. K., Wu, L., Liu, G. Y., Rao, G. H. & Chen, X. L. (2004). *J. Solid State Chem.* **177**, 3454–3459.

[bb9] Rigaku (2005). *CrystalClear* and *NUMABS* Rigaku Corporation, Tokyo, Japan.

[bb10] Sheldrick, G. M. (2008). *Acta Cryst.* A**64**, 112–122. 10.1107/S0108767307043930 18156677

[bb11] Wang, G. F., Fu, P. Z. & Wu, Y. C. (2000). *J. Synth. Cryst* **29**, 130–133.

[bb12] Westrip, S. P. (2010). *J. Appl. Cryst.* **43**, 920–925.

[bb13] Wu, Y. C. & Wang, G. F. (2001). *J. Cryst. Growth*, **229**, 205–207.

[bb14] Yilmaz, A., Bu, X. H., Kizilyalli, M., Kniep, R. & Stucky, G. D. (2001). *J. Solid State Chem.* **156**, 281–285.

